# A superficial esophageal cancer in an epiphrenic diverticulum treated by endoscopic submucosal dissection

**DOI:** 10.1186/s12876-017-0649-y

**Published:** 2017-08-07

**Authors:** Kuangi Fu, Peng Jin, Yuqi He, Masanori Suzuki, Jianqiu Sheng

**Affiliations:** 1 Department of Gastroenterology, Kanma Memorial Hospital, 2-5, Nasushiobara city, Tochigi 325-0046 Japan; 2 Department of Gastroenterology, PLA Army General Hospital, Beijing, 100700 China

**Keywords:** Epiphrenic diverticulum, Superficial esophageal cancer, Magnifying endoscopy, Narrow band imaging, Endoscopic submucosal dissection

## Abstract

**Background:**

We report a unique case of a superficial esophageal cancer arising in a single diverticulum, diagnosed with magnifying image-enhanced endoscopy and then successfully treated by endoscopic submucosal dissection (ESD).

**Case presentation:**

A 66-year-old man with alcohol-related liver injury visited our hospital for endoscopy for investigation of varix. Esophagogastroduodenoscopy showed no varix but a large epiphrenic diverticulum with an area of fainted redness just above the esophagogastric junction. Narrow band imaging revealed a sharply demarcated brownish dotted area, and dilated intra-epithelial papillary capillary loops (IPCL) were subsequently seen after magnification. Chromoendoscopy with 1% Lugol’s iodine solution demonstrated a well-demarcated unstained area, approximately 20 mm in diameter. Endoscopic biopsy revealed a squamous cell carcinoma (SCC).

**Conclusion:**

The tumor was completely resected by ESD without perforation. Histologically, it was an intraepithelial SCC without lympho-vascular invasion of cancer cells. No local recurrence or metastasis was detected at the last follow-up of 42 months.

## Background

Cancer can arise from the normal mucosa near or within an esophageal diverticulum. However, cancer located within a diverticulum is a very rare phenomenon; only sporadic cases have been reported to date. The incidence has been reported to be between 0.3 and 3% [1]. Almost all cases were diagnosed at an advanced stage, treated by surgery or radiation, and with overall poor prognosis. Rarely cases are detected in early stage and surgical resection is favored as diverticula have a characteristically thin wall and endoscopic resection carries a real risk of perforation [2]. Herein, we describe a case of a superficial esophageal cancer developed in an epiphrenic esophageal diverticulum, diagnosed with magnifying image-enhanced endoscopy and subsequently treated by endoscopic submucosal dissection (ESD).

## Case presentation

**Fig. 1:**
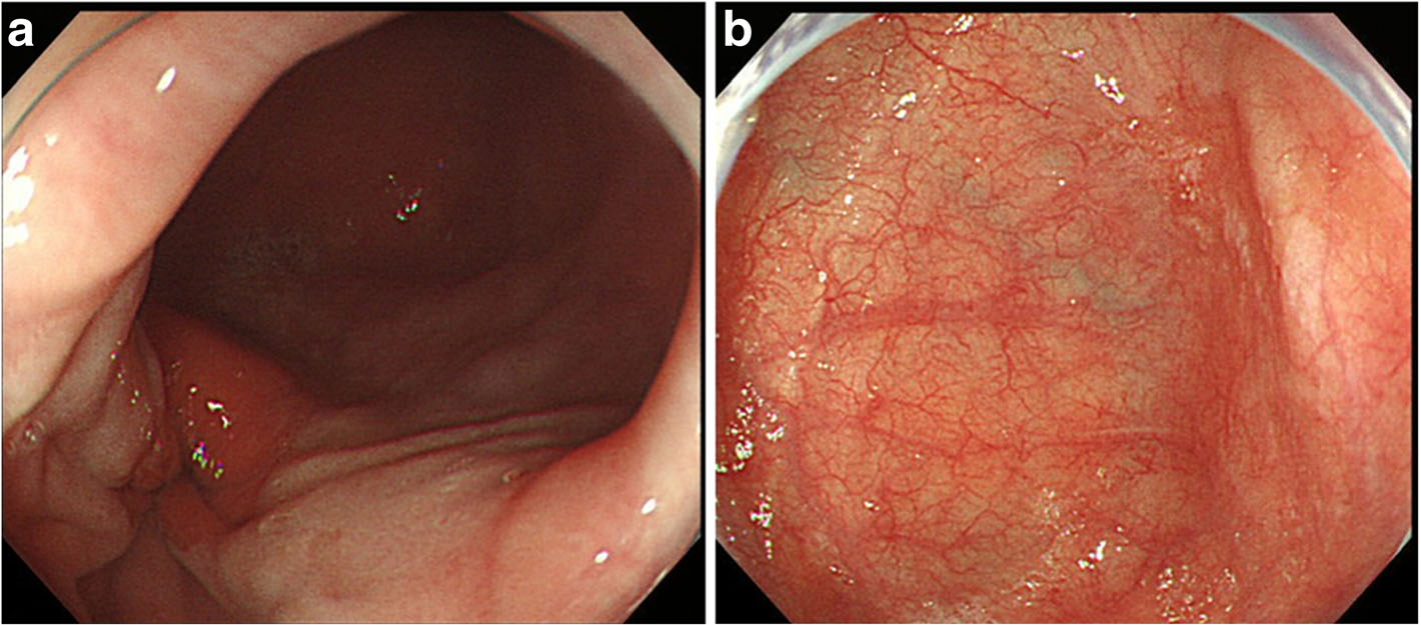
**a** A large epiphrenic diverticulum just *above* the esophagogastric junction was seen during esophagogastroduodenoscopy. **b** An area of fainted redness was detected with white light endoscopy at the bottom of the diverticulum

**Fig. 2:**
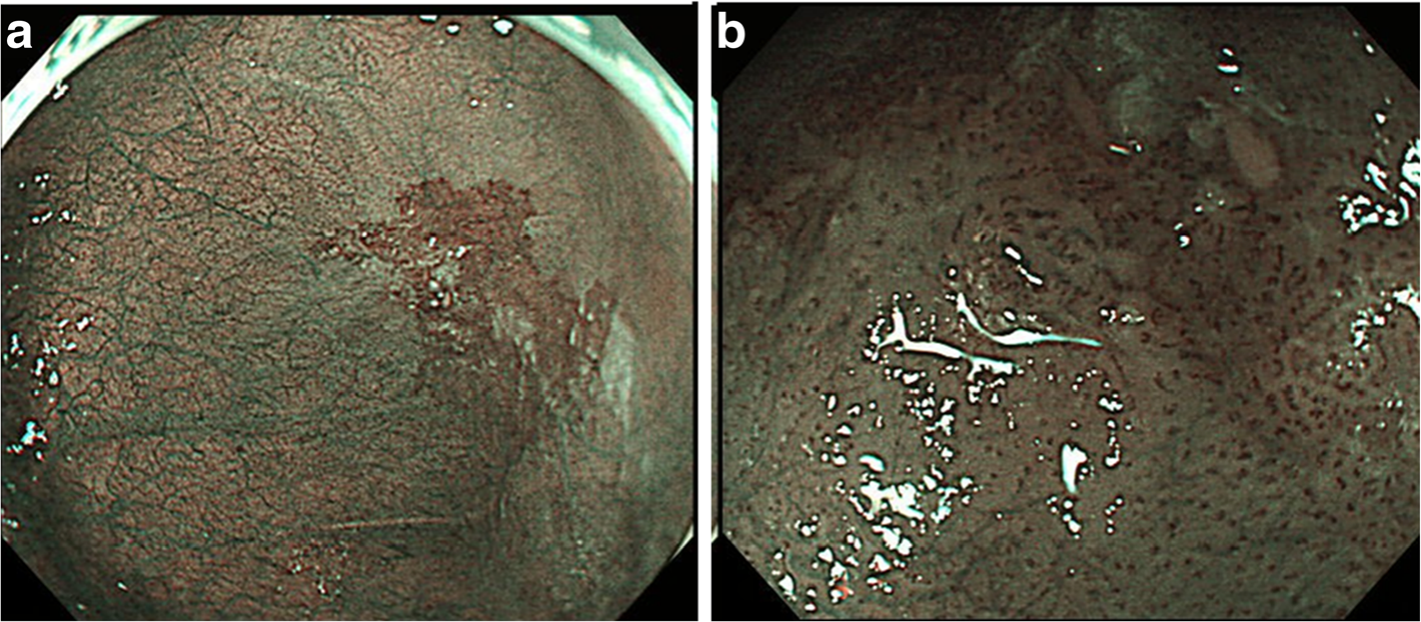
**a** NBI revealed a demarcated *brownish dotted* area before magnification. **b** Dilated intra-epithelial papillary capillary loops (IPCL) were seen after magnifying NBI

**Fig. 3:**
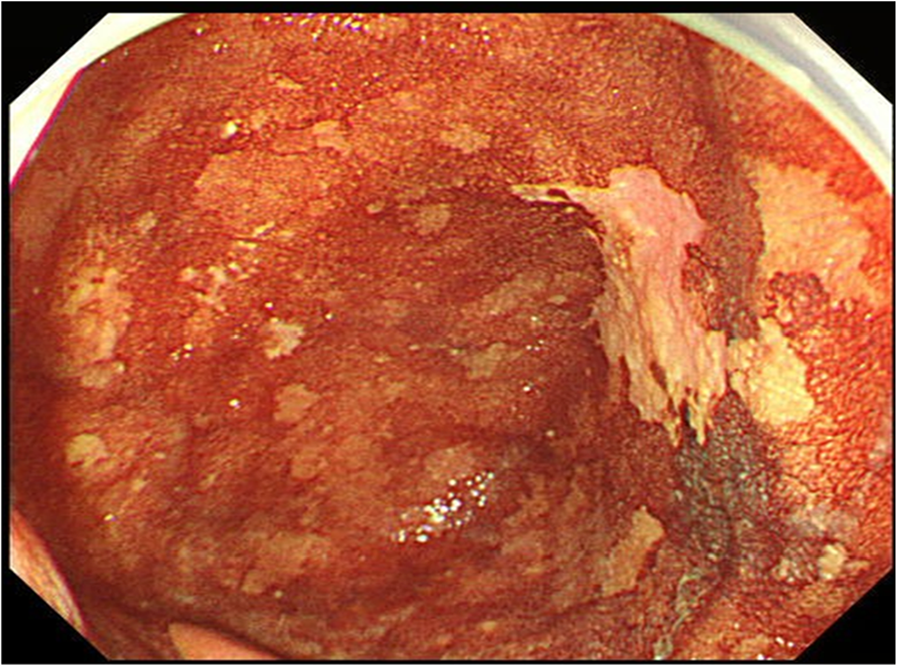
At the *bottom* of the diverticulum, chromoendoscopy with 1% Lugol’s iodine solution demonstrated a well-demarcated unstained area, approximately 20 mm in diameter

**Fig. 4:**
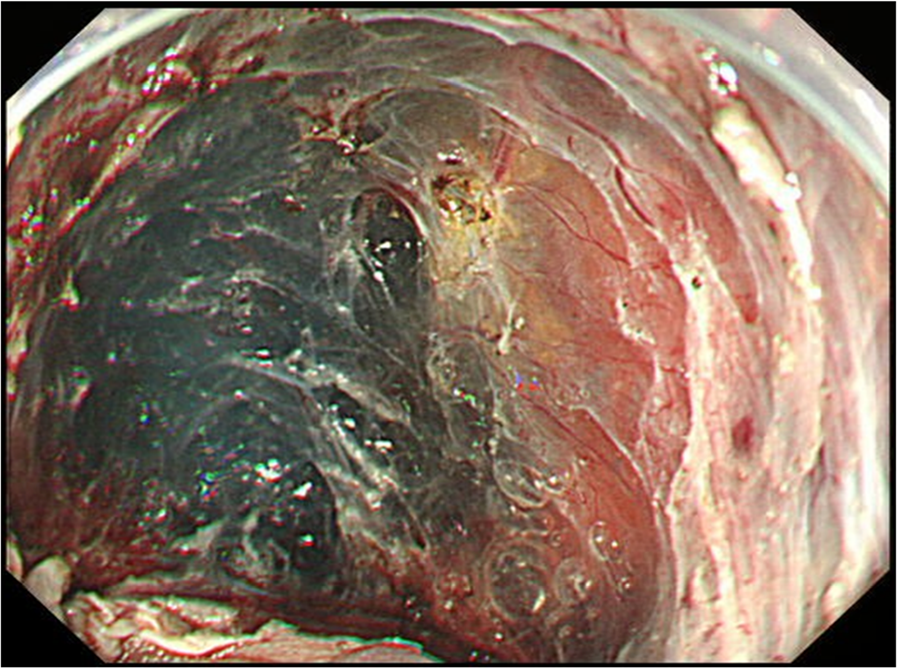
Mucosal defect after ESD was shown and no definite perforation was seen endoscopically

**Fig. 5:**
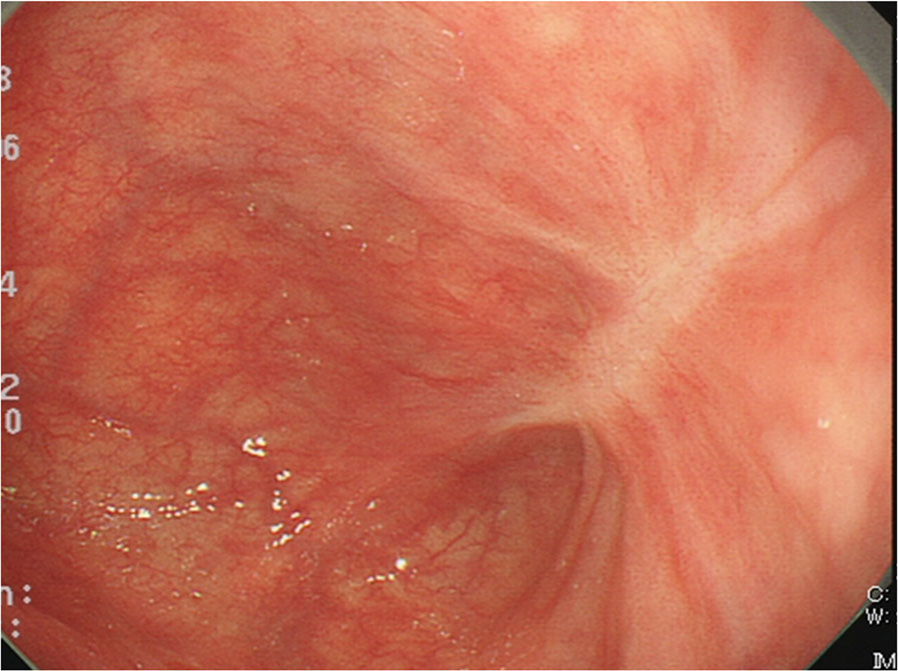
*White light* endoscopy showed no local recurrence 42 months after ESD

A 66-year-old man with alcohol-related liver injury visited our hospital for endoscopy for investigation of varix. Esophagogastroduodenoscopy (Olympus; Japan) showed no varix but a large epiphrenic diverticulum just above the esophagogastric junction (Fig. 1a). An area of fainted redness was detected with white light endoscopy at the base of the diverticulum (Fig. 1b). Narrow band imaging (NBI) endoscopy (GIF-H260Z, Olympus) revealed a demarcated brownish dotted area (Fig. 2a). Dilated intra-epithelial papillary capillary loops (IPCL) classified as B1 according to Japan Esophageal Society classification were subsequently seen on magnification (Fig. 2b) [3, 4]. Chromoendoscopy with 1% Lugol’s iodine solution demonstrated a well-demarcated unstained area, approximately 20 mm in diameter, corresponding to the reddish area (Fig. 3). Pink color sign was also seen in the unstained area about 2 min after iodine staining [5]. Endoscopic biopsy revealed a squamous cell carcinoma (SCC). The depth of invasion of the detected tumor was estimated to remain within the mucosal epithelium (m1) or the lamina propria (m2), which carries almost no potential for nodal involvement. Therefore, ESD was proposed as an alternative to radical surgery and patient consent was obtained. Endoscopic submucosal dissection was conducted with the patient under general anesthesia. CO2 was used for insufflation to decrease the risk of mediastinal emphysema or pneumothorax in the event of perforation. The lesion was well-lifted after submucosal injection of hyaluronic acid diluted with 10% glycerol at the ratio of 50%. The tumor was completely resected en bloc without complication (Fig. 4). Histologically, the resected specimen was a superficial cancer limited within the lamina propria (m2) without lympho-vascular invasion or marginal involvement of cancer cells. The patient had an uneventful hospital course and was discharged 1 week after ESD. No local recurrence or metastasis was detected at the last follow-up of 42 months (Fig. 5).

## Discussion and conclusions

Cancers arising within esophageal diverticulum may be diagnosed at advanced stage despite their small size. As the muscular coat of a diverticulum is extremely thin or none, cancer arising within an esophageal diverticulum can easily extend into the mediastinal space relative to those arising from the normal mucosa apart from the diverticulum. This case was easily detected with the help of magnifying image-enhanced endoscopy in its early stage [3]. Our case illustrates the importance of meticulous endoscopic evaluation of depth invasion of esophageal cancers before removal, as surgery may be avoided in some cases. The changes in the IPCL pattern observed by magnifying NBI were reported to be useful for the qualitative diagnosis of cancerous/non-cancerous lesions and endoscopic diagnosis of invasion depth of cancers [6, 7]. Here we performed endoscopic resection of the lesion, as magnifying image-enhanced endoscopy provided an endoscopic diagnosis of a superficial cancer limited within the lamina propria (m2). It is commonly accepted that esophageal cancers limited within m2 are extremely rarely associated with lymph node metastasis and therefore are good candidates for endoscopic resection [8].

Endoscopic ultrasonography (EUS) is commonly used for predicting the depth of tumor invasion in patients with superficial esophageal squamous cell carcinoma [9]. We did not utilize EUS in this case as part of the diagnostic workup, as it was difficult to appropriately approach the lesion located at the base of the diverticulum. Furthermore, diverticulum has a characteristically thin wall, which may be associated with higher risk of perforation during EUS [10].

Endoscopic mucosal resection (EMR) might be an alternative for local resection. There are three representative methods of EMR: endoscopic esophageal mucosal resection (EEMR)-tube method, EMR using a cap-fitted endoscope (EMRC) method and two-channel EMR method. Generally, the incidence of perforation is lower than that of ESD. However, the lesion described here was not amenable to EMR as pulling the lesion back for resection would have resulted in frank perforation, likely 10 mm or larger in size, making endoscopic closure technically very difficult. Meanwhile, perforation during ESD is always smaller and linear, as the submucosal layer could be dissected under direct visualization [11]. To avoid undesirable perforation, we therefore planned to discontinue ESD if non-lifting sign positive was seen after appropriate submucosal injection. To achieve an appropriate submucosal dissection plane under the tumor for complete removal, mucosal incision and submucosal dissection were started from the oral side of the normal mucosa outside of the diverticulum as both of the submucosal and muscular layers of the diverticulum were expected to be much thinner than those of the normal esophagus histologically.

In conclusion, we report a case of a superficial esophageal cancer developing within an epiphrenic diverticulum. The lesion was correctly diagnosed with magnifying image-enhanced endoscopy and subsequently treated by ESD with long-term success.
